# Systematic Assessment of Chemokine Signaling at Chemokine Receptors CCR4, CCR7 and CCR10

**DOI:** 10.3390/ijms22084232

**Published:** 2021-04-19

**Authors:** Herman D. Lim, J. Robert Lane, Meritxell Canals, Martin J. Stone

**Affiliations:** 1 Infection and Immunity Program, Monash Biomedicine Discovery Institute, Clayton, VIC 3800, Australia; herman.d.lim@gmail.com; 2 Department of Biochemistry and Molecular Biology, Monash University, Clayton, VIC 3800, Australia; 3 Division of Physiology, Pharmacology and Neuroscience, School of Life Sciences, Queen’s Medical Centre, University of Nottingham, Nottingham NG7 2UH, UK; rob.lane@nottingham.ac.uk (J.R.L.); meritxell.canals@nottingham.ac.uk (M.C.); 4 Centre of Membrane Protein and Receptors, Universities of Birmingham and Nottingham, Nottingham NG7 2UH, UK

**Keywords:** chemokine, chemokine receptor, CCR4, CCR7, CCR10, GPCR, signaling pathways, partial agonism, biased agonism

## Abstract

Chemokines interact with chemokine receptors in a promiscuous network, such that each receptor can be activated by multiple chemokines. Moreover, different chemokines have been reported to preferentially activate different signalling pathways via the same receptor, a phenomenon known as biased agonism. The human CC chemokine receptors (CCRs) CCR4, CCR7 and CCR10 play important roles in T cell trafficking and have been reported to display biased agonism. To systematically characterize these effects, we analysed G protein- and β-arrestin-mediated signal transduction resulting from stimulation of these receptors by each of their cognate chemokine ligands within the same cellular background. Although the chemokines did not elicit ligand-biased agonism, the three receptors exhibited different arrays of signaling outcomes. Stimulation of CCR4 by either CC chemokine ligand 17 (CCL17) or CCL22 induced β-arrestin recruitment but not G protein-mediated signaling, suggesting that CCR4 has the potential to act as a scavenger receptor. At CCR7, both CCL19 and CCL21 stimulated G protein signaling and β-arrestin recruitment, with CCL19 consistently displaying higher potency. At CCR10, CCL27 and CCL28(4-108) stimulated both G protein signaling and β-arrestin recruitment, whereas CCL28(1-108) was inactive, suggesting that CCL28(4-108) is the biologically relevant form of this chemokine. These comparisons emphasize the intrinsic abilities of different receptors to couple with different downstream signaling pathways. Comparison of these results with previous studies indicates that differential agonism at these receptors may be highly dependent on the cellular context.

## 1. Introduction

Chemokines (chemotactic cytokines) and their receptors are the key regulators of leukocyte recruitment in both normal immune function and numerous inflammatory diseases [1,2]. Chemokine receptors are expressed on different types of leukocytes and signal in response to chemokine binding, giving rise to regulation of leukocyte adhesion and chemotaxis to sites of high chemokine expression. The chemokine ligands and chemokine receptors interact in a complex network, such that most chemokines activate multiple receptors and most receptors respond to multiple chemokines. In humans, there are ~43 chemokines and ~19 receptors, which belong to the family of G protein-coupled receptors (GPCRs) that signal via heterotrimeric G proteins and non-G protein effectors, such as β-arrestins. Typically, G protein activation via chemokine receptors promotes chemotaxis of the receptor-expressing cells. In contrast, several “decoy” chemokine receptors have been identified that are not coupled to G proteins but still interact with β-arrestins, leading to internalization of the chemokine receptor-chemokine ligand complex (thereby acting as chemokine sinks) [3].

In some cases, different chemokines can activate the same receptor but elicit distinct signaling responses. Such differences are classified as biased agonism if the ligands differ in their relative efficiencies of signaling via two alternative pathways, e.g., G protein versus β-arrestin pathways, or pathways mediated by different G protein subtypes. Biased agonism has been reported for several chemokine receptors, including CCR1, CCR2, CCR4, CCR5, CCR7, CCR10, CXCR2 and CXCR3 [4,5,6,7,8,9]. Biased agonism is distinct from differences in intrinsic efficacy (or partial agonism), which applies to ligands that activate the same pathways but differ in the relative magnitude of the signaling outcomes. For example, we have previously characterized partial agonism at the chemokine receptor CCR2 [10].

Among the chemokine receptors previously reported to exhibit biased agonism are CCR4, CCR7 and CCR10, all of which play key roles in trafficking of T cells, thus contributing to normal T cell selection, immune responses to viral infection, and/or allergic or auto-immune reactions. Each of these receptors has two cognate chemokine ligands [1]. CCR4 activation by CCL17 and CCL22 is primarily thought to regulate the migration of T helper type 2 (T_h_2) cells during the process of T cell selection as well as during allergic responses (e.g., asthma and atopic dermatitis) but may also be involved in recruitment of regulatory T cells and certain subsets of monocytes and dendritic cells (DCs) [11,12]. CCR7 is expressed on various types of T cells, B cells and DCs that migrate to (or within) lymphoid organs, in which the two CCR7 ligands, CCL19 and CCL21, are expressed [13]. CCR10 is expressed on both T and B cells. Activation of CCR10 by CCL27 promotes the recruitment of T lymphocytes to the skin, whereas CCR10 activation by CCL28 stimulates trafficking of immunoglobulin A antibody-secreting plasma cells (B lymphocytes) to mucosal tissues, including intestines, lung, nasal cavity, uterus and lactating mammary gland [14].

A number of previous studies have investigated the possibility that cognate chemokines differentially activate CCR4, CCR7 and CCR10, with potential implications for regulation of lymphocyte trafficking and selection. In the case of CCR4, CCL22 was observed to be more effective than CCL17 at inducing receptor internalization [15,16] and activation by the two ligands was affected differently by receptor mutations or inhibitory antibodies [17]. Similarly, for CCR7, several studies have found that CCL19 was more effective than CCL21 at inducing receptor internalization and β-arrestin recruitment [7,8,9,18,19,20,21]. However, these studies came to different conclusions regarding whether the two ligands stimulate equivalent G protein-mediated responses via CCR7 [7,8,9,21]. In the only study investigating differential agonism at CCR10, Rajagopal et al. [4] reported that both CCL27 and CCL28 effectively stimulated G protein-mediated signaling, whereas only CCL27 was able to simulate β-arrestin recruitment. Considering the apparent importance of differential agonism at these chemokine receptors in regulation of immune cell function, we were interested in identifying the molecular features of both the chemokines and receptors that contribute to these differences in receptor activation. As a prelude to such studies, we undertook a systematic evaluation of differential activation of CCR4, CCR7 and CCR10 by their cognate chemokine ligands. Herein, we report our findings and discuss both similarities and differences amongst these receptors and compared to previous reports.

## 2. Results

### 2.1. Cognate Chemokines Stimulate β-Arrestin Recruitment at CCR4, CCR7 and CCR10

**Figure 1:**
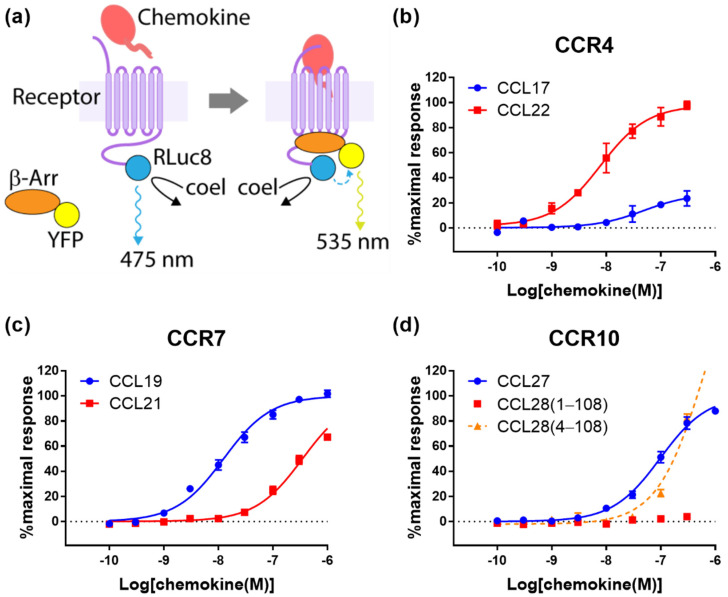
Chemokine ligands differentially stimulate β-arrestin recruitment to CCR4, CCR7 and CCR10. (**a**) The principle of the BRET-based β-arrestin recruitment assay. The *Renilla* luciferase (RLuc8) is fused at the C-termini of the receptors and the yellow fluorescent protein (YFP) tagged at the N-terminus of β-arrestin2 (β-Arr). Upon activation by a chemokine agonist, YFP-β-arrestin2 is recruited to the receptor, bringing the YFP and RLuc8 into close proximity and enabling the resonance energy transfer after addition of the RLuc8 substrate coelenterazine h (coel). (**b**–**d**) Chemokine-induced β-arrestin recruitment at (**b**) CCR4, (**c**) CCR7, and (**d**) CCR10. A dashed line of best fit is shown in the case that the signal does not reach saturation. The presented data are mean ± SEM of at least three independent experiments, each performed in triplicate.

Activation of chemokine receptors typically induces G protein receptor kinase (GRK)-mediated phosphorylation of the receptor C-terminal region and recruitment of β-arrestins to the phosphorylated receptor, leading to receptor internalization [22]. To detect β-arrestin recruitment to CCR4, CCR7 and CCR10, we used Chinese hamster ovary (CHO) cells transiently transfected to express each receptor and β-arrestin2, tagged with a bioluminescence resonance energy transfer (BRET) donor and acceptor, respectively (Figure 1a). Treatment of these cells with cognate chemokines of the expressed receptors stimulated concentration-dependent β-arrestin2 recruitment (Figure 1b–d).

For β-arrestin recruitment to CCR4, CCL22 stimulated a 3.5-fold higher maximal response (*E_max_*) than CCL17 (Figure 1b and Table 1). CCL22 also appeared to have higher potency than CCL17 (*pEC_50_* values of 8.1 ± 0.1 and 7.3 ± 0.3, respectively), although this was not significant.

At CCR7, both CCL19 and CCL21 stimulated robust β-arrestin recruitment responses. However, the potency was approximately 20-fold higher for CCL19 (*pEC_50_* values of 7.9 ± 0.1 and 6.7 ± 0.1, respectively, assuming the same *E_max_* value) and the CCL21 response was not saturated at the highest concentration tested (Figure 1c and Table 1).

At CCR10, CCL27 exhibited robust β-arrestin recruitment (Figure 1d and Table 1). We also tested the two commercially available forms of CCL28. Like other chemokines, CCL28 is initially expressed with an N-terminal signal peptide, which is then proteolytically removed to yield the mature chemokine. Due to the ambiguity of predicting signal peptide cleavage sites, CCL28 is available from different suppliers as the “full-length” form, CCL28(1-108), or a form truncated by three N-terminal residues, CCL28(4-108). We observed that CCL28(1-108) does not significantly stimulate β-arrestin recruitment at CCR10, whereas the shorter form, CCL28(4-108), gave rise to robust, concentration-dependent β-arrestin recruitment (Figure 1d and Table 1). However, since the CCL28(4-108) response was not saturated at the highest concentration tested, we cannot determine whether its potency or maximal response is significantly different from that of CCL27.

### 2.2. Cognate Chemokines Stimulate G Protein-Mediated Signaling at CCR7 and CCR10 But Not at CCR4

**Figure 2:**
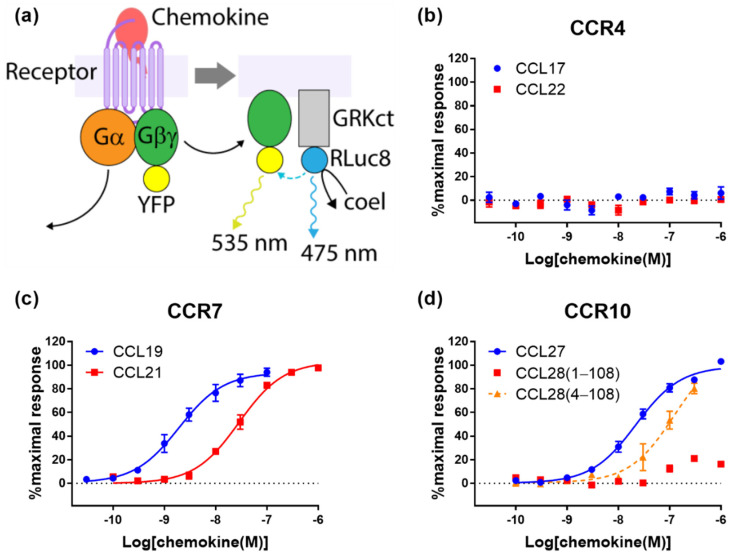
Chemokine ligands differentially stimulate G protein activation via CCR4, CCR7 and CCR10. (**a**) The principle of the BRET-based G-protein recruitment assay. RLuc8 is fused at the C-terminus tail of GRK3 (GRKct), which is localized to the plasma membrane, and the YFP variant Venus is tagged at the Gβ_1_γ_2_ dimer. Upon activation of a chemokine receptor, the activity of the trimeric G protein complex (Gαi2 was overexpressed in this assay) can be detected as the dissociation of Gαi and Gβγ; the latter binds to the GRK3 C-terminal tail, bringing the YFP and RLuc8 into close proximity and enabling resonance energy transfer after addition of coelenterazine h (coel). (**b**–**d**) Chemokine-induced G-protein activation at (**b**) CCR4 (**c**) CCR7, and (**d**) CCR10. A dashed line of best fit is shown in the case that the signal does not reach saturation. The presented data are mean ± SEM of at least three independent experiments, each performed in triplicate.

**Figure 3:**
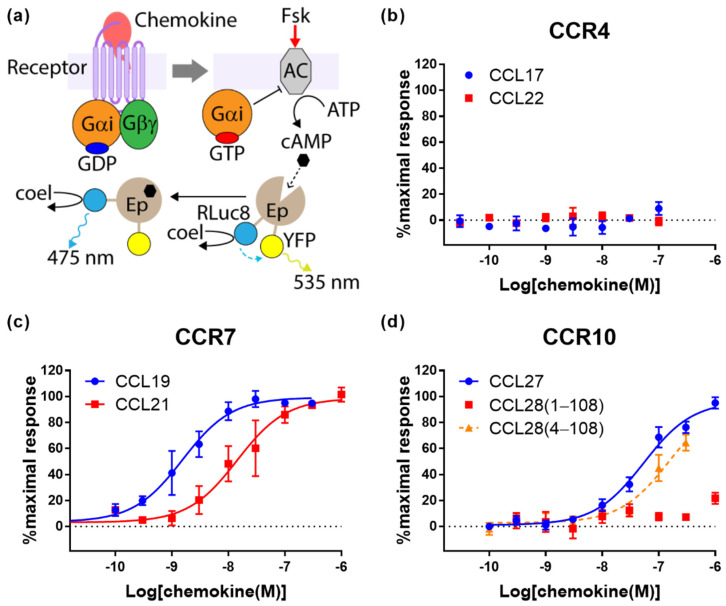
Chemokine ligands differentially inhibit cAMP synthesis via CCR4, CCR7 and CCR10. (**a**) The principle of the BRET-based cAMP assay. Upon activation of a chemokine receptor, the dissociated Gαi/o proteins inhibit the activity of forskolin (Fsk)-stimulated adenylyl cyclase (AC), thus reducing the intracellular concentration of cAMP. The cAMP biosensor CAMYEL is composed of the cAMP binding protein Epac1 (Ep), tagged at opposite ends with RLuc8 and the YFP variant Venus. The conformation of Epac1, within CAMYEL, changes upon binding cAMP and this is used to monitor cellular levels of cAMP by the change of BRET signals. (**b**–**d**) Chemokine-induced inhibition of cAMP production via (**b**) CCR4 (**c**) CCR7 (**d**) CCR10. A dashed line of best fit is shown in the case that the signal does not reach saturation. The presented data are mean ± SEM of at least three independent experiments, each performed in triplicate.

Upon binding their cognate ligands, most chemokine receptors undergo conformational changes that result in dissociation of the Gα subunit from the Gβγ subunit of heterotrimeric G proteins and downstream suppression of cAMP synthesis catalysed by adenylyl cyclase. For real-time measurement of these processes, we used a FlpIn-CHO cell line stably transfected to express the receptor of interest and then transiently transfected with BRET biosensors to report on G protein activation by the receptor (Figure 2a) or inhibition of forskolin-induced cAMP synthesis (cAMP inhibition, Figure 3a).

In CCR7-expressing cells, CCL19 and CCL21 both stimulated robust responses in the assays of G protein activation (Figure 2c and Table 1) and cAMP inhibition (Figure 3c and Table 1). In these assays, both ligands induced similar maximal responses, but CCL19 was more potent by ~15-fold for G protein activation and ~9-fold for cAMP inhibition. This is similar to the relative effects of these ligands in β-arrestin recruitment at CCR7 (Figure 1c).

At CCR10, CCL27 and CCL18(4-108) both exhibited robust G protein activation (Figure 2d and Table 1) and cAMP inhibition (Figure 3d and Table 1). Again, the CCL18(4-108) signals were not saturated at the highest concentrations tested, precluding a rigorous comparison of potencies, although CCL18(4-108) appears to be less potent than CCL27 in this assay. Similar to our observations in the β-arrestin recruitment assay (above), we found that CCL28(1-108) stimulates only very weak G protein activation and cAMP inhibition. Thus, removing the first three amino acids of CCL28 substantially increases both the potency and efficacy (maximum signal) of CCR10 activation in all three signalling readouts.

In stark contrast to the results for CCR7 and CCR10, both cognate chemokines of CCR4 failed to stimulate significant responses in assays of either G protein activation (Figure 2b and Table 1) or cAMP inhibition (Figure 3b and Table 1). To ensure that this was not due to lack of receptor expression, we took advantage of the N-terminal cMyc-tag on all receptors used in this study to determine their expression levels by ELISA (Table 2). Relative cell surface expression levels of CCR4, CCR7 and CCR10 in the stable FlpIn-CHO cell lines used for G protein activation and cAMP inhibition assays were 1:2:30. Although, the FlpIn-CHO-CCR4 cell line has the lowest receptor expression level among the cell lines used here, the level was well above background and only ~2-fold lower than the CCR7 expression level. This slightly lower receptor expression is unlikely to account for the complete lack of G protein-mediated signalling observed. Nevertheless, to investigate this, we constructed a naturally occurring CCR4 that is truncated after residue Y331 (CCR4ΔC29 [23]. This mutant has been reported to display higher cell surface expression and gain of function in a chemotaxis assay [24,25]. As expected, FlpInCHO cells stably expressed N-terminally cMyc-tagged CCR4ΔC29 at a level 25-fold higher than full-length CCR4, as assessed by ELISA (Table 2). Despite its higher cell surface expression, CCR4ΔC29 was not responsive to up to 100 nM CCL17 or CCL22 in either G protein activation or cAMP inhibition assays (data not shown). We therefore conclude that, when expressed in CHO cells, CCR4 is capable of signalling via β-arrestin but not via G proteins.

**Figure 4:**
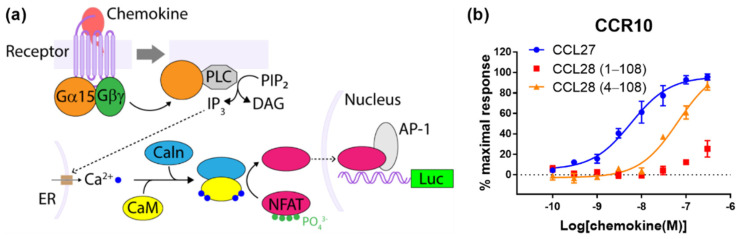
Activation of CCR10 stimulates intracellular Ca^2+^ mobilization. (**a**) The principle of the NFAT activation assay. Activation of a chemokine receptor leads to phospholipase C (PLC) activation via the transiently expressed Gα_15_, giving rise to downstream release of Ca^2+^ from the endoplasmic reticulum (ER). Binding of Ca^2+^ to calmodulin (CaM) then enables activation of the protein phosphatase calcineurin (Caln), which catalyzes dephosphorylation of NFAT, enabling its translocation into the nucleus and transcription of the reporter firefly luciferase gene from an NFAT-sensitive reporter construct. (**b**) Chemokine-induced NFAT activation via CCR10. The presented data are mean ± SEM of at least three independent experiments, each performed in triplicate.

We also evaluated the signalling of CCR4, CCR7 and CCR10 using a reporter gene assay for Nuclear Factor of Activated T Cells (NFAT), which is activated as a consequence of increased cytosolic Ca^2+^ concentrations (Figure 4a). For this we co-transfected the cells to express Gα_15_, which has previously been shown to couple to a variety of GPCRs originally coupled to Gαs or Gαi, along with the NFAT reporter gene construct [26]. Treatment of the CCR10-expressing cells with CCL27 or CCL18(4-108) yielded robust responses (Figure 4b and Table 1), although the CCL27 response was approximately 10-fold more potent than that of CCL28(4-108) (*pEC_50_* values of 8.24 ± 0.12 and 7.18 ± 0.10, respectively, assuming the same maximal effect). Once again, CCL28(1-108) stimulated only a weak response at the highest concentrations tested. Moreover, treatment of the CCR4- or CCR7-expressing cells with the cognate chemokines of these receptors did not yield any significant response above background in the NFAT reporter assay (data not shown).

Finally, we quantified biased agonism at CCR7 and CCR10 using the Black and Leff operational model [27,28]; this was not possible for CCR4 due to the lack of G protein-mediated signaling. None of the possible comparisons provided significant evidence of biased agonism between ligands and pathways mediated by either CCR7 or CCR10 (data not shown). Indeed, this result is consistent with the observation that the relative potencies and efficacies of the different ligands at each receptor are similar, irrespective of the signaling readout.

## 3. Discussion

We have presented a systematic analysis of chemokine signaling at the chemokine receptors CCR4, CCR7 and CCR10. We have shown that, in the same expression system, the three receptors mediate different arrays of signaling outcomes. CCR10 was observed to mediate G protein activation, leading to both cAMP inhibition (via Gα_i/o_) and Ca^2+^ mobilization (via overexpressed Gα_15_), as well as mediating β-arrestin recruitment. CCR7 could mediate the same downstream outcomes as CCR10 with the exception of Ca^2+^ mobilization. In contrast, stimulation of CCR4 did not give rise to any G protein-mediated signaling but clearly induced β-arrestin recruitment.

At each receptor, the ligands tested were not equivalent in the signaling responses that they induced. However, the nature of these differences varied amongst the receptors and ligands studied. The CCR4 ligands differed primarily in their maximal effect (efficacy) of β-arrestin recruitment. The CCR7 ligands differed primarily in their potency in all signaling outcomes tested. The CCR10 ligand CCL27 gave robust signals in all assays, whereas CCL28(1-108) gave very weak signals, although the truncated form, CCL28(4-108), gave similar responses to CCL27, with slightly lower potency in most assays. Moreover, in some cases, the ligand-selective effects contrast with those previously reported for the same receptor and ligands. Below, we discuss the details of these effects for each receptor and attempt to rationalize any inconsistencies between studies.

Our observations that, in all assays, CCL19 displayed higher potency but similar efficacy compared to CCL21, are consistent with previous observations. Early studies reported that, when compared at the same concentration (100 nM), CCL19 was more effective than CCL21 in inducing internalization of CCR7 [19,20]. Similarly, Zidar et al. reported that, in transfected human embryonic kidney (HEK293) cells, CCL19 promoted stronger β-arrestin recruitment than CCL21, although they also found that the two ligands stimulated equivalent G protein-mediated responses [7]. Initially, this appeared to suggest biased agonism by these ligands. Indeed, the same authors found that receptor phosphorylation was mediated by different sets of GRKs, which suggests alternative receptor conformations were favored by the two ligands. However, the signaling experiments of Zidar et al. used a single concentration (100 nM) of each ligand. Our data clearly show that, at this concentration, CCL19 elicits much stronger β-arrestin recruitment than CCL21 (Figure 1c) but both ligands elicit maximal responses in G protein-mediated readouts (Figure 2c and Figure 3c). This results from the β-arrestin signal being essentially unamplified, whereas the G protein-mediated signals are somewhat amplified, causing a left-shift of the concentration-response curves [10]. Additionally, consistent with our data, Corbisier et al. found that CCL19 was more potent than CCL21 in both G protein activation and β-arrestin recruitment (in HEK293 cells), with no significant bias between these ligands and pathways [9]. Two recent studies by Rosenkilde and colleagues have identified features of both the chemokines and CCR7 that contribute to the higher potency of CCL19 [8,21]. In particular, the higher potency of CCL19 was suggested to result from differential interactions of the core domains of the two chemokines with extracellular loop 2 of the receptor [8].

Our CCR10 activation data show that “truncated” CCL28(4-108) robustly activates CCR10, whereas “full-length” CCL28(1-108) has minimal activity in all four signaling readouts tested (Figure 1d, Figure 2d, Figure 3d and Figure 4b). This raises the question of which form(s) of CCL28 is/are biologically relevant. To the best of our knowledge, there are no available data reporting on the naturally occurring N-terminal sequence or molecular mass of CCL28. However, the SignalP program [29] predicts cleavage of the CCL28 precursor at the sequence SEA-ILP (‘-‘ indicates the cleavage site) to yield CCL28(4-108) with 68% probability, compared with cleavage at the sequence LHA-SEA to yield CCL28(1-108) with only 21% probability. Based on this prediction and our activity data, we propose that the active biological form is CCL28(4-108), whose N-terminal sequence, up to CC motif, is ILPIASSCC, and that future physiological studies of CCL28 activity mediated by CCR10 should focus on the shorter form of CCL28. We note, however, that CCL28 has also been reported to activate the receptor CCR3 [30] and that the form(s) of CCL28 active at this receptor remain(s) to be determined.

Unlike the CCR7 observations, our CCR10 activation data differ substantially from a previous report. Rajagopal et al. reported that CCL27 stimulated both G protein-mediated and β-arrestin-recruitment via CCR10, whereas CCL28 stimulated only G protein-mediated signaling; they reported using commercial CCL28(4-108) [4]. This appeared to be a clear example of biased agonism and raised the possibility that differential signaling plays a part in the selectivity of CCL27 and CCL28 for CCR10-expressing T and B lymphocytes, respectively. Our data confirm that CCL27 stimulates both signaling pathways, but differ from the previous report in that we found that CCL28(4-108) also stimulates both G protein-mediated and β-arrestin recruitment via CCR10 (Figure 1d, Figure 2d and Figure 3b), with no evidence of biased agonism between these pathways. We note that the differences in potency observed for CCL27 in the different assays are as expected, due to the different levels of signal amplification/effector coupling in these assays.

On the other hand, we observed a clear difference between the potencies of CCL27 and CCL28(4-108) in the Ca^2+^-sensitive NFAT reporter assay, suggesting subtle bias between different G protein mediated pathways. We note that this apparent bias may be exaggerated because the NFAT assay is highly amplified in comparison to the other assays used here.

Previous studies have found differences in the responses of CCR4-expressing cells to its two cognate ligands. Activation with CCL22, but not with CCL17, has been reported to desensitize CCR4-expressing murine pre-B cells to subsequent activation by either chemokine [15] and induce rapid internalization of CCR4 in human T_h_2 cells [16]. Consistent with these differences, Ajram et al. observed, using the proprietary DiscoverX^®^ cell line, that CCL22, but not CCL17, induced β-arrestin recruitment to CCR4, at chemokine concentrations up to 100 nM [6]. We found that CCL17 does induce β-arrestin recruitment to CCR4 expressed in CHO cells, albeit relatively weakly, at concentrations of 100 nM or higher (Figure 1b). This is in agreement with Viney et al., who reported that 100 nM CCL17 induced significant internalization of CCR4 in the human T cell line Hut78 [17]. These relatively subtle differences in responses to CCL17 may simply reflect the variety of cell types, receptor expression levels and assay formats used in these different studies.

However, there are more substantial differences in the G protein-mediated responses to CCR4 activation observed across different studies. Imai et al. [15] showed robust Ca^2+^ mobilization in response to both CCL17 and CCL22 in CCR4-transfected murine pre-B (L1.2) cells. They also observed chemotaxis of L1.2-CCR4 and Hut78 cells, with CCL22 inducing higher maximal response than CCL17. GPCR-mediated Ca^2+^ mobilization and chemotaxis are generally known to be mediated by G protein signaling, although Lin et al. demonstrated that β-arrestin 2 also plays a partial but important role in CCR4-mediated chemotaxis of murine T_h_2 cells [31]. Subsequently, Viney et al. [17] reported that CCL17 and CCL22 displayed equal potency and efficacy in assays of both Ca^2+^ mobilization (in T_h_2 cells) and chemotaxis (in Hut78 cells or L1.2-CCR4 cells). While CCR4-mediated G protein signaling is apparent in the abovementioned leukocyte cell lines, our results suggest that CCR4 expressed in CHO cells is unable to transmit signal via G proteins. The differences in G protein-mediated responses could be due to expression of different isoforms of G proteins in different cell types. Furthermore, CCR4-mediated G protein activity has been detected in isolated membrane preparations from CCR4-expressing CHO cells, using a [^35^S]GTPγS binding assay [6,32]. This suggests that CHO cells may express soluble factors that suppress G protein signaling by CCR4 in intact cells.

Irrespective of the mechanisms underlying the selective signaling of CCR4 via β-arrestin in CHO cells, our results show that, in certain cellular contexts, CCR4 is capable of supporting β-arrestin recruitment but not G protein-mediated signaling. This effect is reminiscent of atypical chemokine receptors, which lack the DRY sequence motif (which is present in CCR4) and therefore do not couple efficiently to G proteins but still support β-arrestin-mediated events, leading to internalization and scavenging of chemokine ligands [33]. Such scavenging behavior has also been reported for other classical chemokine receptors, such as CCR1 and CCR2 [34,35,36]. Therefore, we now suggest that CCR4 should be viewed as a versatile receptor whose G protein-mediated (pro-chemotactic) and β-arrestin-mediated (scavenging) functions vary with different cellular contexts and could potentially be regulated by such factors as intracellular binding partners, membrane environment, and post-translational modifications.

Finally, it is noteworthy that stimulation of some chemokine receptors, including both CCR4 and CCR7, has previously been reported to induce not only recruitment of β-arrestins leading to receptor internalization, but also downstream signaling via β-arrestin-mediated pathways. Specifically, Lin et al. showed, by genetic deletion of β-arrestin2, that CCL22-induced, CCR4-mediated chemotaxis of Th2 cells was dependent, in part, on a signaling pathway mediated by β-arrestin2 [31]. In addition, Zidar et al. showed, using β-arrestin siRNA in HEK293 cells expressing CCR7, that optimal pERK signaling was dependent on β-arrestin2 expression but opposed by β-arrestin1 expression [7]. The current study did not investigate β-arrestin-mediated signaling per se.

In summary, our systematic study of G protein signaling and β-arrestin recruitment by cognate chemokines at CCR4, CCR7 and CCR10 highlights significant differences between the intracellular signals elicited. At CCR7, both CCL19 and CCL21 stimulate both G protein-mediated and β-arrestin-signals, with CCL19 consistently displaying higher potency. At CCR10, CCL27 and CCL28(4-108), but not CCL28(1-108), stimulate G protein-mediated and β-arrestin-signals, suggesting that CCL28(4-108) is the biologically active form of this chemokine. Finally, at CCR4, both CCL17 and CCL22 selectively activate β-arrestin-mediated pathways, with CCL22 stimulating a substantially stronger signal, suggesting that CCR4 has the potential to act as a scavenger receptor. Comparisons of our results with previous studies further indicate that differential agonism at these receptors may be highly dependent on the cellular context.

## 4. Materials and Methods

### 4.1. Materials

FlpInCHO cells were purchased from Thermo Fisher Scientific. Dulbecco’s modified Eagle’s medium (DMEM), fetal bovine serum (FBS), penicillin/streptomycin, hygromycin B, and D-luciferin were obtained from Life Technologies (Carlsbad, CA, USA). Coelenterazine h was purchased from Prolume (Pinetop, AZ, USA). Enzymes and other materials for molecular cloning were sourced from New England Biolabs (Ipswich, MA, USA). Linear 25 kDa polyethyleneimine (PEI) was from Polysciences (Warrington, PA, USA). The human chemokines CCL17, CCL19, CCL21, CCL22, CCL27 and CCL28 (1-108) were supplied by Peprotech (Cranbury, NJ, USA) and human CCL28(4-108) was from BioLegend (San Diego, CA, USA). White 96-well CulturPlates were purchased from PerkinElmer (Boston, MA, USA). All other reagents were purchased from Sigma-Aldrich (St. Louis, MO, USA).

### 4.2. Cell Lines Stably Expressing Human CCR4, CCR7, and CCR10

The cDNAs encoding the human CCR4, CCR7, and CCR10 were obtained from the cDNA Resource Center (www.cDNA.org). A nucleotide sequence encoding C-myc peptide (MEQKLISEEDL) followed by GGATCCGGA (encoding peptide sequence GSG, and including a *Bam*HI site) was added to the 5′ end of the cDNAs and cloned into a modified pcDNA5/FRT/TO vector (the CMV promoter of the original vector is replaced with the EF1α promoter from pEF4).

FlpInCHO cells were maintained in DMEM supplemented with 5% FBS and 100 u/mL penicillin and 100 μg/mL streptomycin. For stable transfection, the transfection mixture (4.5 μg pOG44, 0.5 μg plasmid containing the receptor construct, and 30 μg PEI in 0.5 mL 150 mM NaCl) was added into a 10-cm dish containing four million adherent cells. One day after transfection, the cells were diluted and selected with 200 μg/mL hygromycin B.

### 4.3. β-Arrestin Recruitment Assay

The DNAs encoding *Renilla* luciferase variant 8 (RLuc8) tagged at the C-termini of CCR4, CCR7, and CCR10 were constructed by PCR amplifying the C-myc-tagged chemokine receptor cDNAs mentioned above and cloned into a pcDNA3.1-derived plasmid containing the gene encoding Rluc8 [37]. The DNA encoding yellow fluorescent protein (YFP) tagged at the N-terminus of the rat β-arrestin2 [37] was obtained from Prof. Kevin Pfleger.

The C-myc-CCR4-RLuc8, C-myc-CCR7-RLuc8, and C-myc-CCR10-RLuc8 genes were transiently co-expressed with YFP-β-arrestin2 in FlpInCHO cells by transfection (4.5 μg receptor-Rluc DNA + 0.5 μg YFP-β-arrestin2 DNA in a 10-cm dish containing four million cells). One day after transfection, the cells were transferred into a white 96-well plate at a density of 50,000 cells/well in 100 μL medium and incubated overnight. The cells were stimulated in Hank’s balanced salt solution (HBSS) in the presence of chemokine at the indicated concentrations and 5 μM coelenterazine-h, with a total assay volume of 80 μL. The BRET signals were measured at 10 min after stimulation, using a PHERAstar microplate reader (BMG Labtech, Ortenberg, Germany) to detect the fluorescence at the wavelengths 450–500 nm and 515–560 nm.

### 4.4. G Protein Activation Assay

FlpInCHO cells stably expressing the chemokine receptors were transiently transfected to express the biosensors for G-protein activation, including Gα_i2_, Gβ_1_-venus (containing amino acid 156–239 of venus), Gγ_2_-venus (containing amino acid 1–155 of venus), and mas-GRK3ct-RLuc8 [38]. One day after transfection, the cells were transferred into a 96-well plate, stimulated and BRET was detected as described above for the β-arrestin recruitment assay.

### 4.5. cAMP Inhibition Assay

FlpInCHO cells stably expressing the chemokine receptors were transiently transfected to express the biosensors for cAMP, called CAMYEL [39]. One day after transfection, the cells were transferred into a 96-well plate, stimulated and BRET was detected as described above for the β-arrestin recruitment assay.

### 4.6. NFAT Activation Assay

FlpInCHO cells stably expressing the chemokine receptors were transiently transfected to express Gα_15_ (obtained from the cDNA Resource Center) and the NFAT-firefly luciferase reporter gene. The transfer of the transfected cells into white 96-well plates performed the same way as described above. The cells were stimulated for six hours with several concentrations of chemokines, diluted in DMEM at a total volume of 100 μL/well. The medium was discarded and firefly luciferase activity was measured in 25 μL luciferase reagent (0.8 mM ATP, 230 μg/mL D-luciferin, 18 mM MgCl_2_, 77 μM Na_2_H_2_P_2_O_7_, 24 mM Tris–H_3_PO_4_ (pH 7.8), 24% (v/v) glycerol, 1.6% (v/v) Triton X-100, and 527 μM dithiothreitol) using a PHERAstar microplate reader (BMG Labtech, Ortenberg, Germany) at 15 min after the addition of the reagent.

### 4.7. Enzyme-Linked Immunosorbent Assay (ELISA)

The FlpInCHO cells stably expressing the chemokine receptors were transferred to a 48-well plate (100,000 cells/well in growth medium) and incubated overnight. The cells were washed, fixed with 4% formaldehyde (in phosphate-buffered saline, PBS) for 30 min, incubated with blocking buffer (PBS in the presence of 1% bovine serum albumin (BSA)) for 1 h, and then exposed to 400 ng/mL monoclonal 9E10 anti-Myc (CSIRO, Melbourne, Australia) for 2 h. The cells were washed two times before treated for 1 h with HRP-conjugated rabbit anti-mouse antibody (Sigma Aldrich) that was 1000-fold diluted in PBS supplemented with 1% BSA. After washing out the secondary antibody, the enzymatic reaction was performed using SigmaFast^TM^ OPD (Sigma Aldrich) and terminated with 3 M HCl. The A_492_ was measured in a PHERAstar microplate reader (BMG Labtech, Ortenberg, Germany).

### 4.8. Data Analysis and Statistics

The BRET signal presented in this manuscript is defined the ratio of fluorescence signals measured at 515–560 nm to those measured at 515–560 nm. For data normalization, 0% is defined as the activity of a receptor in the absence of any chemokine and 100% is the maximal effect (E_max_) of CCL22, CCL19, or CCL27 at the relevant receptor (CCR4, CCR7, or CCR10, respectively), with the exception for CCR4 in G protein activation and cAMP inhibition assays. For these latter cases, 0% is defined as the activity of a receptor in the absence of any chemokine and the maximal effect (E_max_) of CCL27 at CCR7 is used as a surrogate 100%. The concentration vs. response data were plotted and analyzed with the standard single slope dose–response mathematical model of GraphPad Prism 8.0 (GraphPad Software, CA, USA).

## Figures and Tables

**Table 1:** Potencies and maximal effects of cognate chemokines at CCR4, CCR7 and CCR10 ^1^.

Receptor	Assay	Ligand	Potency *pEC_50_* ± SE ^1^ (*EC_50_ in nM*)	Maximal Effect (%) *E_max_* ± SE ^1^
CCR4	β-arrestin recruitment	CCL17	7.31 ± 0.32 (48)	28 ± 6 **
		CCL22	8.11 ± 0.10 (8)	100
	G protein activation	CCL17	ND	ND
		CCL22	ND	ND
	cAMP inhibition	CCL17	ND	ND
		CCL22	ND	ND
CCR7	β-arrestin recruitment	CCL19	7.93 ± 0.06 (12)	100
		CCL21	6.45 ± 0.03 (352) ***	100 (^2^)
	G protein activation	CCL19	8.71 ± 0.09 (2)	100
		CCL21	7.54 ± 0.05 (29) ***	103 ± 3
	cAMP inhibition	CCL19	8.81 ± 0.12 (2)	100
		CCL21	7.84± 0.16 (14) *	99 ± 6
CCR10	β-arrestin recruitment	CCL27	7.01 ± 0.06 (97)	100
		CCL28(1-108)	ND	ND
		CCL28(4-108)	ND	82 (^3^)
	G protein activation	CCL27	7.66 ± 0.05 (22)	100
		CCL28(1-108)	ND	ND
		CCL28(4-108)	6.92 ± 0.14 (121)	80 (^3^)
	cAMP inhibition	CCL27	7.27 ± 0.10 (54)	100
		CCL28(1-108)	ND	ND
		CCL28(4-108)	6.82 ± 0.23 (152)	64 (^3^)
	NFAT Ca^2+^ reporter	CCL27	8.24 ± 0.12 (6)	100
		CCL28(1-108)	ND	ND
		CCL28(4-108)	7.18 ± 0.10 (65) **	106 ± 8

^1^*pEC_50_* values are the negative log of *EC_50_*, in molar units. The corresponding *EC_50_* values (in nM) are shown in parentheses. *E_max_* values are relative to the reference ligand (CCL22, CCL19, or CCL27 at CCR4, CCR7, or CCR10, respectively). ND, not determinable from the experimental data (see Figure 1, Figure 2 and Figure 3). Data are mean ± SEM of at least three independent experiments, performed each in triplicate. * *p* < 0.05, ** *p* < 0.01, *** *p* < 0.001, relative to the reference ligand at the same receptor. Statistical analysis was performed using one-way ANOVA with Holm-Sidak’s multiple-comparison. ^2^ This *E_max_* value was constrained to be the same as that of the reference ligand; the corresponding *pEC_50_* value is influenced by this assumption. ^3^ These *E_max_* values listed are the maximum signals observed as the curves did not reach saturation.

**Table 2:** Expression levels (%) of chemokine receptors CCR4, CCR7 and CCR10 in CHO cells ^1^.

Receptor	Normalized Expression Level
CCR4ΔC29 CCR4	100 4.2 ± 1.2
CCR7	7.9 ± 2.0
CCR10	120 ± 24

^1^ Cell surface expression levels of cMyc-tagged chemokine receptors in stably transfected FlpIn-CHO cells were determined by cMyc ELISA. Reported levels (mean ± SEM, *n* ≥ 3) are arbitrarily normalized as percentage to the corresponding level of C-terminally truncated (29 amino acid residues) CCR4 mutant (CCR4ΔC29), determined in parallel.

## Abbreviations

ANOVA	analysis of variance
BRET	bioluminescence resonance energy transfer
BSA	bovine serum albumin
cAMP	3’,5’-cyclic adenosine monophosphate
CCL	C-C motif chemokine ligand
CCR	C-C motif chemokine receptor
CHO	Chinese hamster ovary
CXCR	C-X-C motif chemokine receptor
DC	dendritic cell
DMEM	Dulbecco’s modified Eagle’s medium
E_max_	maximal response
ELISA	enzyme-linked immunosorbent assay
GPCR	G protein-coupled receptor
pEC_50_	log_10_(*EC_50_*) where *EC_50_* is the concentration (in molar) required for 50% activation
NFAT	nuclear factor of activated T cells
PBS	phosphate-buffered saline
PEI	polyethyleneimine
PLC	phospholipase C
RLuc8	Renilla luciferase 8
T_h_2	T helper type 2
YFP	yellow fluorescent protein
